# Trends and factors associated with intimate partner sexual violence among married women in Zambia: A multilevel analysis

**DOI:** 10.1371/journal.pone.0318640

**Published:** 2025-03-31

**Authors:** Simona Simona, Nakena Likando

**Affiliations:** Department of Social Work and Sociology, School of Humanities and Social Sciences, University of Zambia, Lusaka, Zambia; University of Bergen: Universitetet i Bergen, NORWAY

## Abstract

**Background:**

Sexual violence against women is a major social and public health problem with wide-ranging consequences on the victims. In Zambia, 15% of women have experienced sexual violence from intimate partners. While there exist some studies on sexual violence in Zambia, there is a dearth of research specifically addressing underlying determinants of intimate partner sexual violence against women who are either married or living with partners. Our study sought out to examine factors associated with sexual violence among this demographic and how these factors vary across communities and over time.

**Method:**

We used data from the 2007, 2013/14 and 2018 waves of the Zambia Demographic and Health Surveys (ZDHS) consisting of a total of 21,020 women of reproductive age group of 15-49 years old. Trends in intimate partner sexual violence by selected factors across the three ZDHS cycles were analyzed using bivariate statistics and multilevel logistic regression models. Multilevel models were also fitted on the pooled data, including all the survey years, to examine individual and contextual level factors associated with intimate partner sexual violence.

**Results:**

The results show that the prevalence of sexual violence remain high in Zambia, with a slight decrease over time, from 16.3% in 2007 through to 14.7% in 2018. Trends analysis show that the prevalence rate among women who have attained higher education, are in the highest wealth quintile, reside in urban areas, have decision-making authority have decreased significantly between 2007 and 2018. Contrarily, among women who tolerate violence and those who live with alcohol consuming partners, sexual violence prevalence rates have increased over time. In the consolidated multilevel model, the study finds several factors to be associated with intimate partner sexual violence, including having primary education (AOR =  1.18; 95% CI 1.02-1.36), having an alcohol consuming partner (AOR =  1.76; 95% CI 1.61-1.93), having no decision-making authority (AOR =  1.19; 95% CI 1.09-1.31), witnessing parental violence (AOR =  1.47; 95% CI 1.34-1.60), having a controlling partner (AOR =  3.41; CI 2.96-3.92) and being in a polygynous relationship (AOR =  1.31; 95% CI 1.16-1.49). While women who occupy the highest wealth quintile (AOR =  0.72; 95% CI 0.57-0.90) and those who live in rural areas (AOR =  0.83; 95% CI 0.72-0.96) have lower odds of experiencing intimate partner sexual violence.

**Conclusion:**

This study underscores the persistent prevalence of sexual violence against women in Zambia, particularly within the context of intimate relationships. While there has been a slight decrease in prevalence over time, there are significant disparities across demographic and socio-economic factors. Findings indicate that educational attainment, partner’s controlling behavior, decision making authority, exposure to parental violence, partner’s alcohol consumption, and relationship type significantly influence the likelihood of experiencing intimate partner sexual violence in Zambia. These findings imply that there is need for targeted policy interventions and strategies to address underlying determinants of intimate partner sexual violence and promoting gender equality and empowerment among women in Zambia.

## Introduction

The United Nations defines violence against women as “any act of gender-based violence that results in, or is likely to result in, physical, sexual, or mental harm or suffering to women, including threats of such acts, coercion or arbitrary deprivation of liberty, whether occurring in public or in private life” [1]. Sexual violence is a form of gender-based violence and is defined as “any sexual act or an attempt to obtain a sexual act, unwanted sexual comments, or advances, acts to traffic or otherwise directed, against a person’s sexuality using coercion, by any person regardless of their relationship to the victim in any setting, including but not limited to home and work” [2]. Article 2 of the 1993 UN Declaration on the Elimination of Violence against Women identifies many different forms of violence against women and these include; battering, infanticide, sexual abuse, marriage rape, sex trafficking, genital mutilation and dowry-related abuse among others [1]. The World Health Organization (WHO) estimates that about 35.6% of women have experienced sexual violence [3]. The form and type of sexual violence against women, however, differs from society to society and from culture to culture [4,5]. In Zambia, approximately one in five women between the age of 15 and 49 years were life-time victims of sexual violence. Sexual violence prevalence rate for ever-married women in the same age group stand at 16% [6].

Sexual violence has wide-ranging repercussions for its victims, manifesting in several social and health problems. These include but not limited to depression, chronic pain, homelessness, unwanted pregnancies, substance abuse, suicidal tendencies, unsafe abortions, and pregnancy complications [4,5]. Sexual violence can also contribute to socioeconomic disparities as victims may endure social and economic burdens such a lost income, personal insecurity and productivity [7]. Moreover, sexual violence presents substantial challenges to economically disadvantaged countries like Zambia, as it reduces worker productivity and income, impedes the accumulation of human and social capital as well as exacerbates existing burdens on healthcare and judicial systems [4].

Several factors have been advanced to explain why some women get to be victims of sexual violence while others are protected from it. In sub-Saharan Africa, inequitable gender norms which privilege men with power over women are often shown to be associated with the risk of intimate partner sexual violence [8,9]. This power dynamic tends to be exploited in acts of sexual violence by those who wild it. In such situations, sexual violence against women is either condoned or trivialized, which contributes to a culture of impunity for perpetrators and silence, or victim blame towards survivors [4]. On the other hand, research suggests that protective factors against sexual violence for women include empowerment [10], employment [11], access to financial resources [12], and educational attainment [9]. However, it is important to note that these protective factors may not offer permanent immunity as some studies have reported instances of ‘violence backlash’ wherein men may escalate intimate partner violence in an effort to challenge the perceived shift in women’s status and empowerment [13].

In an effort to address violence against women, Zambia made sexual violence as part of the Zambian Penal Code in 1995 [14] and later in 2011, the country enacted the anti-gender-based violence act (AGVA) [15]. The penal code is focal tool applied by courts against sexual violence perpetrators and prescribes punishment for sexual offences to the maximum terms of life imprisonment to act as a deterrent. The AGVA provided, among other things, a legal framework for the establishment of victim shelters across the country. It is within this framework that the government, together with partner organizations, has operated the ‘One-stop Center’ (OSC) model to provide key services to victims of sexual and gender-based violence. The OSC model is an integrated strategy where key agencies such as the police, health, social workers, and other agencies are brought in one location to provide both legal and social support to victims [16].

The OSCs and other deterrent measures may be responsible for the slight reduction in sexual violence prevalence rates in Zambia over time. However, to sustain and improve concerted efforts of fighting against sexual violence, it is crucial that the underlying factors that are associated with sexual violence are well understood. It is also important to assess how the influence of such factors on sexual violence have evolved over time. This is particularly so in marital relationships where sexual violence may be more prominent because of the existence of cultural norms which socializes women into believing that marriage confers the ‘right’ of sexual access to husbands [17]. Identifying predictors of intimate partner sexual violence against married women is useful in designing targeted programmes and interventions to stop or reduce the problem.

Several studies have been conducted globally to address different dimensions of sexual violence [18–23]. Reviewed literature indicates that no national and dedicated studies on trends and factors associated with sexual violence against married women have been conducted in the Zambian context. This makes it difficult to understand the risk factors of intimate partner violence among this demographic. The current study fills in this void in literature by using nationally representative sample data to identify the factors associated with intimate partner sexual violence among married women, and how these factors vary between communities and over time. The Zambia Demographic and Health Survey (ZDHS) has been implementing the Domestic Violence Module (DVM) and it has been generating data that can be used to assess trends and the influences of different factors on sexual violence. This study uses data from the three cycles ZDHS with DVM data that have been conducted so far (2007, 2013/14, and 2018).

## Materials and methods

### Design and data sources

This is a repeated cross-sectional study and it used secondary data from three cycles of the Zambia Demographic and Health Survey (ZDHS) containing the DVM data including 2007, 2013/14 and 2018. The ZDHS is a nationally representative population-based survey of women and men of reproductive age (15-49 years for women, and 15-59 years for men) designed to provide information on several topics including fertility, family planning, childhood and adult mortality, maternal and child health, HIV/AIDS, and domestic violence among others. The ZDHS has been conducted in Zambia since 1996, with data available for five survey cycles spanning between the years 1996 to 2018 exists. The first two cycles of the ZDHS were excluded from this analysis because the DVM containing relevant variables was not introduced until the 2007 survey.

### Sampling

The ZDHS uses a two-stage cluster sampling by first selecting clusters or primary sampling units (PSUs) and in the second stage households to be interviewed are sampled within selected clusters. All eligible members within the household (women and men of reproductive age group) are interviewed for the survey. The most recent Zambian population census is used as the sampling frame. Further details on the sampling procedure is given in the ZDHS report [6]. Data for the paper were derived from the women’s questionnaire and the analysis was restricted only to women who reported to be marriage, formerly married, or having a partner.

### Variables and variable operationalization

To measure the magnitude of sexual violence, it is important to have operational definitions based upon specific behaviors. Operationalization is the process through which abstract and theoretical concepts are turned into observable and measurable entities. These standard measures help to avoid subjective interpretations and allows for comparability across sites and replicability in future studies. The dependent variable for this study was life-time experience of sexual violence by a male partner. In the ZDHS questionnaire, respondents were asked whether they had ever experienced any sexual violence from an intimate partner. The variable is dichotomous with two possible outcomes of ‘yes’ and ‘no.’

Independent variables in this study were delineated into individual and community-levels to reflect the hierarchical nature of the ZDHS, with individual women nested within clusters or PSUs. The variables were identified on account of their theoretical relevance, dominance in literature, and author’s experience. Using these criteria, variables on women’s socio-demographic characteristics, relationships with their partners as well as previous experience of parental domestic violence were selected. Sociodemographic variables included current age (15-24, 25-34, 35-49), wealth index (first, second, middle, fourth, highest) and educational attainment (no education, primary and higher). Relational factors included husband’s alcohol consumption, decision-making authority within the household, husband’s controlling behavior, wife-beating attitude, and family type.

Husband’s alcohol consumption is a dichotomous variable with ‘yes’ or ‘no’ as possible responses. Decision-making authority is a composite variable from a few questions asking women about the person who usually decides on the respondent’s health care, large household purchases, purchases for daily use, and visits to family and relatives. In this study, a woman who made decisions either independently or in collaboration with a partner in any of the items listed was considered to have decision-making authority, otherwise they didn’t. Husband controlling behavior was determined if the respondent answered in affirmative to any of the following: a) husband is jealousy if she talks with other men b) husband accuses her of unfaithfulness, c) husband doesn’t permit her to meet female friends, d) husband tries to limit contact with family or e) husband always insists on knowing her whereabouts. Wife-beating attitude was measured by questions asking respondents whether wife beating was justified if a wife a) goes out without informing the partner; b) neglects children; c) refuses to have sex; and d) argues with partner. A composite variable that combines answers to all the four questions was created, categorizing it as “yes” or “no”. Family type variable was derived from a question asking respondents to indicate the number of co-wives, and it was recoded as a binary with ‘0’ to denote no other wife and ‘1’ to mean two or more co-wives. Experience of parental domestic violence is a dichotomous variable related to whether the respondent’s father ever beat her mother.

Community-level factors were aggregates of individual level variables at the PSU level because the DHS only collects individual-level data. These included community controlling behavior, community decision-making authority, community polygynous marriage, and community wife-beating attitude. Community variables were recoded as low and high based on the median value. Place of residence is an original ZDHS variable which can explain characteristics of clusters directly with two categories of ‘rural’ or ‘urban’.

### Statistical analysis

All data extractions, management and analysis were done using the R statistical programming environment [24]. Specifically, we used the *dplyr* package [25] for data management and *lme4* package [26] for multilevel analysis. Data analysis was done in three cycles. Firstly, we used bivariate descriptive analysis and chi-squared tests to determine trends in intimate partner sexual violence against women across the three ZDHS cycles and associations between each of the independent variables and the dependent variable. Bivariate analyses were applied to assess whether temporal relationships exist between the outcome variable and each of the independent variables and how these relationships change over time. All bivariate descriptive analysis were based on weighted frequencies and proportions to restore representativeness of the data and make correct estimates of population parameters.

Secondly, we applied multilevel logistic regression models on each of the three datasets to produce both random and fixed effects for sexual violence in relation to selected individual and community-level factors. This is in view of the hierarchical nature of the ZDHS with individual women nested within PSUs, which violates the assumption of independence associated with standard logistic regression. To this end, it is expected that women in the same PSU (community) have similar characteristics compared to women from other PSUs. This phenomenon produces clustering, which suggests the need to examine between cluster variability using the variance partition coefficient (VPC) available in multilevel analysis. Besides, we wanted to assess how the effects of community-level variables as well as the relationships between selected independent variables and experiences of intimate partner sexual violence have evolved over time.

Thirdly, multilevel logistic regression models were applied on pooled data from three surveys to assess the relative effects of selected individual and community-level factors on intimate partner sexual violence in Zambia. The VPC and adjusted odds ratios (AOR) with corresponding 95% confidence intervals (CI) were reported for this purpose. In this analysis, multilevel logistic models were estimated. Model 1 was the null model, including only the intercept and the outcome variable. Model 2 included only individual-level variables while Model 3 included both the individual and community-level variables. The multilevel model assesses the probability $p_{ij}$ of a woman *i* in a community *j* experiencing sexual violence. The analysis is represented as:


$$logit(p_{ij})=\beta_{0}+\beta {Χ}_{ij}+uj+\upsilon$$ (1)

where $X_{ij}$ is a vector of independent variables at individual and community-levels. uj is normally distributed with variance $\sigma_{u}^{2}$; *υ* is normally distributed with variance $\sigma_{u}^{2}$.

The VPC was used to assess the extent to which variations in experiences of sexual violence was attributable to community-level factors relative to individual-level variables. VPC provides information on the share of variance at each level. The latent method was used to calculate the VPC at each level. It assumes a threshold model, approximating the level 1 variance by $\pi^{2}/3\left(\approx 3.29\right)$ [27,28]. The community level variance for example is calculated as follows:


$$VPC_{Community}=\frac{\sigma_{u}^{2}}{\sigma_{u}^{2}+\pi^{2}/3}$$ (2)

The goodness of model fit in the three models was assessed by comparing the Bayesian Information Criteria (BIC) produced in each of the models. Smaller BIC denoting good model fit.

### Ethical consideration

The ZDHS is one of the ICF International-managed surveys and all surveys that involve human participants are approved by ICF institutional review boards and the host country’s ethical review panel. Due to the sensitivity of the DVM in the women’s questionnaire, however, some more stringent measures are applied to insure not only maximum confidentiality and privacy but also the safety of participants. These include special training to field staff who handle this module, obtaining informed consent, conducting the interview in a private place, information regarding options available for those women experiencing domestic violence and collaboration with local women’s groups among others. Further information on ethical procedures is contained in the ZDHS reports: https://bit.ly/3lvJEzc

## Results

### Trends in intimate partner sexual violence against women in Zambia

**Fig 1:**
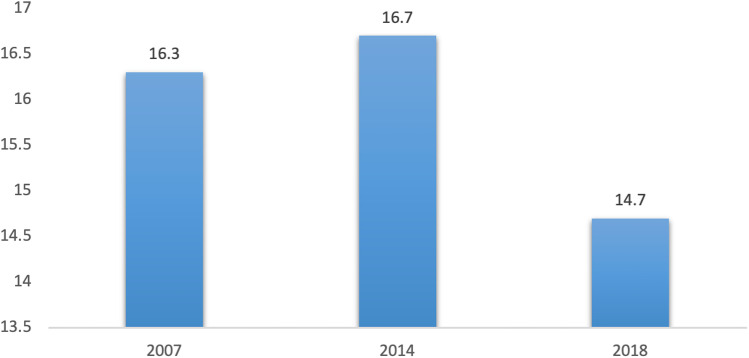
Percentage trends in intimate partner sexual violence against women by survey year.

A total of 21,020 who reported to be ever married or living with a partner in the three ZDHS cycles were included in the final analysis. There are variations in the sample size for the three ZDHS cycles, from 4,246 in 2007, it increased to 9,416 in 2014 but reduced to 7,258 in 2018. The prevalence rate of intimate partner sexual violence against women in Zambia remains relatively high and this phenomenon has largely remained the same over the period of 10 years under observation, between 2007 and 2018. In 2007, women who reported to be victims of intimate partner sexual violence stood at 16.3%. This figure increased slightly to 16.7% in 2014 then decreased to 14.7% in 2018. Fig 1 reports the percentage distribution of intimate partner sexual violence by survey year.

The bivariate analysis indicates that trends in intimate partner sexual violence against women in Zambia, based on selected variables, have remained largely consistent between 2007 and 2018. The relationship between age and the experience of intimate partner sexual violence was not statistically significant over the ten-year period. During this time, the prevalence of intimate partner sexual violence increased and decreased by a similar margin among women aged 15–24 and 35–49 years. In contrast, the prevalence rate among women aged 25–34 remained relatively stable from 2007 to 2013/14, before declining to 14.6% in 2018.

Intimate partner sexual violence has been high across all levels of education and wealth status, with significant relationships over time (p < 0.01). Among those with higher education as well as those belonging to the fourth and highest wealth quintile, the prevalence rate of intimate partner sexual violence significantly decreased between 2007 and 2018. For example, the prevalence rate among women in the highest quintile dropped by eight percentage points, from 17.1% in 2007 to 9.1% in 2018. Among women with no education, those with primary education, second wealth quintile and those in the third quintile, the rate of sexual violence increased slightly in 2014 and then decreased in 2018.

The relationship between area of residence and intimate partner sexual violence was significant in 2007 (p <  0.001 and 2014 (p <  0.01) but ceased to be significant in 2018. Trends show that the prevalence rate of intimate partner sexual violence is decreasing in urban areas, from 19.6% in 2007 to 14.7% in 2018 while in rural areas, the prevalence rate increased from 14.7% in 2007 to 18% in 2014 and then reduced to 14.9% in 2018. As expected, husband alcohol consumption was found to be associated with sexual violence over the years. We found that the prevalence rate of intimate partner sexual violence among women married to men who consume alcohol was high and it seems to increase over time from 19.8% in 2007 to 23.1% in 2018.

Decision-making authority was not significantly associated intimate partner sexual violence in 2007 but the association became significant in 2014 and 2018 ZDHS (p <  0.001). The trend analysis indicates that the prevalence rate among women who have decision-making authority is decreasing over time, from 15.7% in 2007 to 10.4% in 2018 compared to those without decision-making authority. Among women with family violence experience, intimate partner sexual violence prevalence rate was higher, and the relationship seems to be consistently significant over the 10 years under investigation.

Other variables that were found to be significantly associated with intimate partner sexual violence include wife-beating attitude, controlling behavior and family type. Prevalence rates of sexual violence are higher among women who are tolerant of violence and among those married to men with controlling behavior. Similarly, intimate partner sexual violence prevalence rates have been high for women in polygynous marriages in 2014 and 2018 compared to those in monogamous marriages in the same period. The trends in the sexual violence prevalence rates seem to be the same between both types of marriages which saw an increase in 2014 and then a decrease in 2018.

### Percentage distribution of intimate partner sexual violence against women aged 15-49 in Zambia

Table 1 also reports percentages of women who reported to have ever-experienced intimate partner sexual violence from pooled data, combining the three cycles of the ZDHS. For all years combined, the results show that a higher percentage of women with no education and primary education reported to have experienced sexual violence compared to women with women with secondary education and above. The same applies to women in the highest wealth index who reported lower proportions of sexual violence compared to the women in the lower wealth index.

**Table 1:** Trends in percentage distribution of lifetime experience of intimate partner sexual violence by selected characteristics using ZDHS.

Variables	Experiencing intimate partner sexual violence							
	2007 (n = 4246)		2013/14 (n = 9416)		2018 (n = 7358)		Pooled data (21020)	
**Age**	ns		ns		ns		ns	
15 – 24	15.7	1133	16.4	2129	14.1	1768	15.4	5030
25 – 34	17.1	1893	17.6	4155	14.6	2940	16.6	8988
35 – 49	16.2	1220	17.1	3132	14.8	2650	16.0	7002
**Education**	**		***		***		***	
No education	11.6	586	17.9	1073	17.0	825	16.1	2484
Primary	17.8	2562	18.6	5243	16.2	3881	17.6	11686
Higher	16.1	1098	14.5	3093	11.3	2652	13.5	6843
**Wealth**	***		***		***		***	
First	13.8	816	17.8	2081	17.8	1916	17.2	4813
Second	15.2	874	19.0	2045	16.6	1613	17.4	4532
Third	16.0	957	20.0	2072	13.0	1503	16.8	4532
Fourth	19.9	974	15.6	1881	13.1	1286	15.8	4141
Highest	17.1	625	11.4	1337	9.1	1040	11.8	3002
**Residence**	***		**		ns		ns	
Urban	19.6	1532	16.0	3920	13.7	2459	16.0	7911
Rural	14.7	2714	18.0	5496	14.9	4899	16.2	13109
**Husband alcohol consumption**	***		***		***		***	
Yes	19.8	2319	20.2	4462	23.1	2314	21.0	9597
No	12.2	1996	13.6	5085	9.6	4558	11.8	11639
**Decision-making authority**	ns		***		***		***	
Yes	15.7	1557	13.8	4487	10.4	3709	12.5	9874
No	14.6	2059	19.9	3471	18.2	2454	17.9	8124
**Witnessing violence**	***		***		***		***	
Yes	20.1	1963	20.7	4223	19.4	2653	20.2	8839
No	12.9	2274	14.3	5173	11.8	4705	13.1	12152
**Wife beating attitude**	**		***		***		***	
Yes	17.6	2766	19.5	3752	19.5	3752	19.7	11497
No	14.4	1480	9.4	3606	9.4	3606	11.8	9523
**Controlling behaviour**	***		***		***		***	
Yes	19.7	3331	21.0	7032	18.8	5157	20.0	15520
No	4.8	915	5.9	2355	4.6	2201	5.2	5471
**Family type**	ns		***		***		***	
Monogamy	15.1	3110	15.4	7018	12.6	5419	14.4	15547
Polygyny	14.6	506	24.1	936	19.7	711	20.4	2153

**Notes**: *** p <  0.001; ** =  p <  0.01; *  =  p <  0.05; ns =  not significant

A higher proportion of women who live with men who consume alcohol reported to have experienced sexual violence (21%) compared to those who don’t live with such men (11.8%). More women with ‘no’ decision-making authority (17.9%) report being victims of intimate partner sexual violence compared with women who have authority (12.5%). It is also apparent that having history of parental violence is a factor in sexual violence such that women who report to have witnessed violence between parents were more likely to experience intimate partner sexual violence compared to women without a history of parental violence. Moreover, there are significant differences in susceptibility to sexual violence between women who tolerate violence and woman who do not. Women who tolerate violence are more likely to experience sexual violence (19.7%) compared to those who do not (11.8). An even bigger difference exists among women married or living with controlling behavior (20%) compared to women without such partners (5.2%). Furthermore, polygyny does not seem to offer any cushion for sexual violence as the evidence shows that women in monogamous marriages are less likely to experience sexual violence (14.4%) compared to those in polygynous marriages (20.4%).

### Multilevel analysis of factors associated with lifetime experience of intimate partner sexual violence among married women aged 15-49 years

The results for multilevel analysis taking cluster or PSU as level 2 are presented in Table 2 using the odds ratios and 95% confidence intervals (CI) for both the individual and community-level variables. Both random and fixed effects for the three cycles of the ZDHS are displayed with appropriate levels of significance.

**Table 2:** Multilevel regression of factors associated with intimate partner sexual violence in Zambia from 2007 to 2018 using adjusted odds ratio and 95% CIs.

Variables	2007 (n = 4246) AOR (CI)	2013/14 (n = 9416) AOR (CI)	2018 (n = 7358) AOR (CI)
**Education**			
No education	1	1	1
Primary	1.43(1.03,1.98)^*^	1.09(0.88,1.36)	1.09(0.84,1.42)
Higher	1.06(0.71,1.57)	0.92(0.71,1.19)	0.87(0.64,1.18)
**Wealth**			
First	1	1	1
Second	1.14(0.83,1.58)	1.01(0.83,1.24)	0.96(0.77,1.19)
Third	1.04(0.75,1.45)	1.14(0.91,1.42)	0.76(0.58,0.98)^*^
Fourth	1.13(0.74,1.71)	0.91(0.69,1.21)	0.70(0.50,0.99)^*^
Highest	0.98(0.59,1.64)	0.81(0.57,1.16)	0.55(0.36,0.84)^**^
**Husband alcohol**			
No	1	1	1
Yes	1.70(1.39,2.07)^***^	1.62(1.42,1.86)^***^	2.31(1.96,2.73)^***^
**Decision-making authority**			
Alone or with husband	1	1	1
Husband or other	0.85(0.69,1.04)	1.24(1.08,1.42)^**^	1.41(1.19,1.66)^***^
**Witnessing violence**			
No	1	1	1
Yes	1.51(1.24,1.84)^***^	1.53(1.34,1.74)^***^	1.32(1.12,1.55)^***^
**Wife beating attitude**			
No	1	1	1
Yes	1.10(0.88,1.36)	1.41(1.22,1.64)^***^	1.74(1.46,2.08)^***^
**Controlling behaviour**			
Yes	1	1	1
No	4.17(2.94,5.91)^***^	3.52(2.85,4.35)^***^	2.98(2.33,3.81)^***^
**Family type**			
Monogamy	1	1	1
Polygyny	1.02(0.76,1.36)	1.45(1.19,1.76)^***^	1.30(1.04,1.64)^*^
**Contextual variables**			
**Residence**			
Urban	1	1	1
Rural	0.79(0.55,1.13)	0.95(0.76,1.20)	0.71(0.53,0.94)^*^
**Community decision-making authority**	1.01(1.00,1.02)^*^	1.00(1.00,1.01)^*^	1.01(1.01,1.01)^**^
**Community polygynous marriage**	0.99(0.98,1.01)	1.01(1.00,1.02)^*^	1.01(1.01,1.01)^**^
**Random effects**			
Variance	0.31	0.53	0.61
VPC %	8.56	13.87	15.64

Our findings show significant differences in odds of experiencing sexual violence among women with primary education compared to those with no education in 2007. The odds of experiencing sexual violence among women with primary education are 1.43 times more than for women with no education. However, this significant relationship does not hold in the subsequent cycles of the ZDHS. Wealth status shows an interesting picture whereby wealth index does not influence sexual violence significantly prior to 2018. In 2018, there are significantly lower odds of experiencing sexual violence for women in higher wealth quintile compared to those in the first. For example, women in the highest quintile have 0.55 times lower odds of experiencing intimate partner sexual violence compared to those in the first quintile.

Factors which have consistently been associated with intimate partner sexual violence against women across the three survey years include husband’s alcohol consumption, witnessing violence, and controlling behavior. In 2007 women who were married or living together with partners who consume alcohol had 1.7 more odds of experiencing sexual violence compared to those without such partners. The relationship between husband alcohol consumption and experiencing sexual violence was highest in 2018 where the odds were 2.31 times more for women married to alcohol consuming men compared to their counterparts.

The odds of experiencing sexual violence are higher among women who reported to have witnessed parental violence. In the 2007 survey the odds of experiencing violence among such women were 1.51 times higher than women who did not witness violence between parents. There was a slight increase in the 2014 survey, but the odds reduced significantly in the 2018 survey. As expected, higher odds of experiencing sexual violence are observed among women married or living together with men who have controlling behaviors, although the odds are reducing over time, from 4.17 in 2007 to 2.90 in 2018.

Family types is another significant contributing factor to intimate partner sexual violence among women who are ever married in Zambia. Whereby women polygynous in polygynous partnerships have higher odds of experiencing sexual violence compared to their counterparts in monogamous relationships. The effects of family type on sexual violence were not significant in 2007 but became significant in 2014 and 2018. The odds of experiencing intimate partner sexual violence were 1.45 times more for women in polygynous relationships than those in monogamous relationships in 2014 and in 2018, the odds reduced to 1.30.

Place of residence, community decision-making authority and community polygynous marriages were the three community level variables that were associated with intimate partner sexual violence albeit only in the 2018 survey year. It is interesting for example, that there are no significant differences are observed between rural and urban areas regarding intimate partner sexual violence in survey years prior to 2018 where living in rural areas reduces the odds of experiencing sexual violence by 0.71.

Although most of variations in sexual violence were attributed to individual level factors, our analysis shows significant between cluster variability in intimate partner sexual violence were also observed. The variance partition coefficients (VPC) in the null model (before controlling for the effects of any individual or community-level predictors) indicates that about 9% (that is: (0.31)/0.31 + 33.29) of the total variation in sexual violence is attributed to community factors in 2007, with the remaining 91% attributable to individual-level factors. However, the VPC increased in 2014 and 2018 to 14% and 16% respectively. For all the three cycles, there was a reduction in the VPC after the individual and community-level factors were introduced into the models (not reported).

### Multilevel modelling analysis of factors associated with intimate partner sexual violence in pooled ZDHS data

Table 3 reports the results from multilevel analyses of pooled data, where we sought to assess the combined influence of individual and community-level variables on intimate partner sexual violence. It also shows measurements of between cluster variability and the goodness of fit across the three models. This analysis controlled for the effect of survey year.

**Table 3:** Multilevel analysis of factors associated with intimate partner sexual violence with adjusted odds ratio and 95% CI (n =  21020).

Variables	Model 1	Model 2	Model 3
**Intercept**	0.18(0.13,0.23)	0.03 (0.02,0.03)	0.02(0.01,0.03)
**Survey year**			
2007		1	1
2014		1.24(1.10,1.40)^***^	1.24(1.10,1.40)^***^
2018		1.10(0.96,1.25)	1.12(0.98,1.27)
**Education**			
No education		1	1
Primary		1.19(1.03,1.37)^*^	1.18(1.02,1.36)^*^
Higher		0.97(0.82,1.15)	0.95(0.80,1.13)
**Wealth**			
First		1	1
Second		1.04(0.91,1.18)	1.03(0.90,1.17)
Third		0.99(0.86,1.14)	0.98(0.85,1.13)
Fourth		0.88(0.74,1.05)	0.87(0.72,1.04)
Highest		0.73(0.59,0.91)^**^	0.72(0.57,0.90)^**^
**Husband alcohol**			
No		1	
Yes		1.76(1.61,1.92)^***^	1.76(1.61,1.93)^***^
**Decision-making authority**			
Yes		1	1
No		1.23(1.13,1.35)^***^	1.19(1.09,1.31)^***^
**Witnessing violence**			
No		1	1
Yes		1.47(1.35,1.60)^***^	1.47(1.34,1.60)^***^
**Wife beating attitude**			
No		1	1
Yes		1.43(1.30,1.58)^***^	1.43(1.30,1.57)^***^
**Controlling behaviour**			
No		1	1
Yes		3.43(2.98,3.96)^***^	3.41(2.96,3.92)^***^
**Family type**			
Monogamy		1	1
Polygyny		1.36(1.21,1.54)^***^	1.31(1.16,1.49)^***^
**Contextual variables**			
**Residence**			
Urban		1	1
Rural		0.86(0.75,0.99)^*^	0.83(0.72,0.96)^**^
**Community decision-making authority**			1.00(1.00,1.01)^*^
**Community polygynous marriage**			1.02(1.00,1.02)^*^
**Random effects**			
Variance (SE)	0.25	0.24	0.23
VPC %	7.06	6.80	6.53
BIC	18371.7	13930.2	13914.2

Most of the factors that were associated with intimate partner sexual violence across the three cycles have remained so in the pooled analysis. For example, the results show that women married to men with controlling behavior are more than three times as likely to experience sexual violence compared to women who are not married to such partners. Other attributes that seem to strongly expose women to sexual violence include being married to men who consume alcohol, having no decision-making authority, witnessing parental violence, tolerance of violence and being in polygynous marriages. Contrarily, being in the highest quintile of wealth status reduces women’s odds of experiencing sexual violence by 0.72 times, after controlling for the effects of community-level variables.

At the community level, place of residence, community decision-making authority and community polygynous marriages remain to be significantly associated with intimate partner sexual violence. Variations in sexual violence are also found to be mostly due to individual-level factors. Model 1 which only has the intercept shows that community level factors contribute about 7% to the variations in intimate partner sexual violence. Little change in the VPC after introducing individual factors in model 2 and community-level factors in model 3 indicates stronger heterogeneity in intimate partner sexual violence across communities. The BIC values are decreasing as individual and community variables are introduced, which means that model 3, which reports a complete two-level model, is a considerably better predictor of intimate partner sexual violence compared to the first two models.

## Discussion

This study sought out to identify factors associated with intimate partner sexual violence against married women in Zambia, examining how these associations vary across different communities and evolve over time. As such, we firstly, applied multilevel models of the same variables to each of the 2007, 2014 and 2018 ZDHS cycles, and secondly, we applied the same models on pooled data. We focused on two level to measure the amount of variation in intimate partner sexual violence attributable to individual and community level factors. To our knowledge, this is the first comprehensive analysis of the ZDHS data, involving the three survey cycles which implemented the domestic violence module.

Overall, the study found that intimate partner sexual violence against women in Zambia has slightly decreased over the ten-year period under consideration. However, with a prevalence rate of 14.7%, it remains high and unacceptable. Comparable trends have been observed in other African countries, including Ghana [17], Nigeria [29], and Zimbabwe [10,30]. The slight reduction may be attributed to many factors, including the Anti-Gender-Based Violence Act of 2011 [15], that was operationalized in 2013. The Anti-Gender-Based Violence Act of 2011 established a legal framework for the creation of GBV and User-Friendly Fast Track Courts, which are currently operational in most provinces. These courts likely serve as a deterrent to potential perpetrators of sexual violence, contributing to the reduction in prevalence rates.

There are considerable differences in the trends of intimate partner sexual violence among selected variables. While progress in terms of declining prevalence rates is noted among some, other groups of women have recorded increased risk of sexual violence over the years. For instance, a significant increase in sexual violence is observed among women with no education, those in the first wealth quintile, those living with alcohol consuming partners and those who are tolerant of violence. On the other hand, a consistent reduction in likelihoods of experiencing sexual violence is observed among women with higher education, higher wealth quintile, those with decision-making authority as well as women who reside in urban areas.

Factors which are associated with intimate partner sexual violence across the survey years include partner’s alcohol consumption, partner’s controlling behavior and witnessing parental violence. The contribution of decision-making authority and wife-beating attitude on intimate partner sexual violence seem to be gaining ground over time. Explanations for the consistent contribution of these factors to sexual violence have been linked to inequitable gender norms which contribute to unequal power relations between men and women in society [4,9,30]. These findings show the deep-rooted nature of persistent gender norms and social expectation which continue to promote male dominance in the Zambian society.

In the comprehensive model, factors such as primary education, having a partner with alcohol consumption habits, history of parental violence, tolerance for violent behavior, presence of a controlling partner, limited decision-making authority, and engagement in polygynous relationships are identified as significant contributors to increased likelihood of experiencing intimate partner sexual violence. On the other hand, belonging to the highest wealth quintile and residing in rural areas provide protections against intimate partner sexual violence.

The relationship between educational status and sexual violence has been varied in literature. The current study is aligned with those which revealed that educational level is not associated with sexual violence in adjusted models [22,31]. Other studies found that education offers protection against intimate partner sexual violence [18,21,32].

Contrary to some studies in sub-Saharan Africa, which reported no significant relationship between wealth status and sexual violence victimization [17,31], our study found that in Zambia, women in the highest wealth quintile have lower odds of experiencing intimate partner sexual violence. This result is consistent with another study conducted in Zambia [9], and can be attributed to structural and economic inequalities which increase women’s vulnerability to physical, sexual, and emotional abuse, particularly due to their economic dependence on their male partners. It is plausible that the economic autonomy of women is a protective factor for intimate partner sexual violence because on one hand, men may be uncomfortable with the risk of losing the economic value that such women bring into the relationship, and on the other hand, wealthier women have increased agency of reporting cases of abuse to the justice system.

Husband’s alcohol consumption behavior is significantly associated with intimate partner sexual violence. As expected, our results indicate that women living with partners who consume alcohol are 1.76 times more likely to experience intimate partner sexual violence after controlling for the effects of other variables. This finding is supported by studies elsewhere [17,18,21,32–34]. Although determining direct causal connections between alcohol consumption and violence is difficult due to data limitations, it is possible that alcohol could cloud judgement about acceptable norms and values in society leading drunk men to exhibit violent sexual behavior towards their sexual partners. Alcohol may also be used as a socially acceptable justification for husbands abusing their wives over different reasons [34].

The finding that witnessing parental violence in childhood is associated with intimate partner sexual violence is consistent with the life course theories which attempt to make connections between the past and the present [35]. Accordingly, children who have witnessed incidents of parental violence have a high likelihood of becoming aggressors in future intimate partnerships. In a similar manner, women who have witnessed their father abuse their mother in the past, especially if the violence was unpunished, are more likely to internalize and legitimize aggressive behavior and regard it as ‘normal’ in marital relationship [17].

Male dominance is another factor that is very crucial to sexual violence. Our study, like others [17,21,22] found that women who live with husbands who exhibit controlling behavior, and are major decision makers in the households are more likely to experience sexual violence compared to those who live with husbands without such behaviors. Controlling behavior was significantly associated with intimate partner sexual violence in this study to the extent that controlling behavior was associated with over three-fold higher likelihood of sexual violence after controlling for potential confounders. Controlling behavior among men is constructed as a demonstration of unequal power relations between men and women which is characteristic of patriarchal societies. Male partners may engage in sexual violence as a way of displaying control over their opposite sex partners and dominance is based on internalized beliefs about male superiority.

In patriarchal societies like Zambia, women are demanded by culture to submit and respect their husbands as well as condone male superiority and attitudes that may even be harmful to themselves [17]. Wife-beating attitudes that are found to be associated with intimate partner sexual violence in this study can be explained by such cultural background. This is consistent with previous studies in Nigeria [22] and Haiti [36]. Wife beating is often regarded as a demonstration of love by a husband or as a symbol of authority [25,29]. Women who condone violence may do so in conformity with internalized cultural norms which defines women as inferior and privileges men with rights and power to dominate women including the use of force [37].

The relationship between marital status and sexual violence is well documented by most researchers [9,18,38]. Mainly the relationship is such that those who are either married, divorced, or separated are more at risk of sexual violence than those who are never married. This study focused instead on the relationship between family/marriage type and intimate partner sexual violence, and we found that women in polygynous relationships are more likely to experience sexual violence than those in monogamous relationships. Similar findings are observed in Nigeria [22]. This finding can be attributed to jealous, in that partners may fear that their spouses might engaged in extra marital relationships. Such husbands tend to exhibit controlling behaviour and resort to sexual violence as a way of obtaining compliance from their spouses.

Another interesting finding was the relationship between community level factors and intimate sexual violence. Although most of the variations was at the individual level, significant variations in sexual violence were found across communities. It is particularly notable that the significance of this relationship is increasing over time. This could mean that individual behaviors and actions are increasingly influenced by the social context to which individuals belong [39] and this phenomena buttresses the rationale for the use of multilevel models in public health and sexual violence studies.

Place of residence, community decision-making authority, and community polygynous marriages were significantly associated with intimate partner sexual violence at the community level. This study departs from previous studies, some of which failed to find a relationship between place of residence and sexual violence [17] or suggested that urban areas are associated with lower odds of sexual violence [22,31]. However, our study established that residing in rural areas is associated with decreased likelihood of experiencing sexual violence. Discrepancies in these findings may be due to differences in context and the unit of analysis. Nonetheless, the idea that rural residence acts as a protective factor against sexual violence aligns with sociological scholarship portraying rural societies as tightly knit, homogeneous, and characterized by collective consciousness [40]. Consequently, it is plausible that rural communities possess greater influence in shaping social behavior and curbing societal deviance against established norms and values, such as sexual violence.

The relationship between decision-making authority and intimate partner violence has often produced conflicting results depending on the type of decision making and the nature of violence [8,10]. However, our study shows that decision-making authority was associated with decreased odds of intimate partner sexual violence as earlier studies. It has also established that polygynous marriage increases the odds of intimate partner sexual violence. Preferential treatment to some wives over others, which is often characteristic of polygynous marriages can exacerbate the risk of intimate partner violence especially for senior wives [41] Moreover, it has been established that intimate partner violence is more likely to occur in polygynous marriages due to low cooperation among co-wives attributed to heightened competition over scarce resources and to win favors from the husband [42,43].

### Strength and limitations

This study has key strengths due to the use of nationally representative large sample combining ZDHS data from 2007 to 2018 and adjusting for both individual and community level factors. The analysis underscores the value of including community variables or any other contextual factors in the study of intimate partner sexual violence. However, this study is not without limitations that should be considered in relation to the study findings. The cross-sectional study design used in this analysis does not enable any inference of causal directionality between explanatory and outcome variables. The other limitation relates to the ZDHS self-reported data collection procedures which may be disadvantageous for topics like sexual violence which may be considered private matters especially in marriages or intimate relationships. As such, women may be unwilling to disclose certain cases of sexual violence and thus, underestimating the prevalence rates of intimate partner sexual violence as well as it’s risk factors. Moreover, the interpretation of sexual violence may not be uniform across individuals and time. These limitations notwithstanding, the ZDHS is part of international surveys which, have the reputation of producing high quality reliable data because of the rigor in the sampling design, training and supervision of interviewers as well as data processing and analytical procedures [44,45].

## Conclusion

This study attempted to analyze the trends and factors associated with intimate partner sexual violence against women in Zambia using the 2007, 2014 and 2018 cycles of the ZDHS. The findings suggest that despite recording a slight decrease in intimate partner sexual violence between 2007 and 2018, there is still a high prevalence of intimate partner sexual violence in Zambia. Major factors that contribute to intimate partner sexual violence against women across all the survey years include husband’s alcohol consumption, witnessing parental violence, and controlling behavior. Significant variations in the relationships are observed over time. For example, the relationship between partner’s controlling behavior and intimate partner sexual violence is high but reducing over time while that of husband alcohol consumption, wife-beating attitudes and decision-making authority have increased. Overall, educational status, wealth quintile, husband alcohol, decision-making authority, witnessing violence, wife beating attitude and controlling behavior are associated with intimate partner sexual violence. Although most of the variations are attributed to individual level factors, social contexts such as rural residence, living in communities with high proportions of women with decision-making authority and low proportions of community polygynous marriages reduces the odds of intimate partner sexual violence significantly. Therefore, policy interventions and programs aimed at reducing intimate partner sexual violence, should be targeted at both individual and contextual risk factors in order to promote gender equality and empowerment among women in Zambia.

## References

[1] Assembly UG. Declaration on the Elimination of Violence against Women. UN General Assembly. 1993.

[2] Krug EG, Mercy JA, Dahlberg LL, Zwi AB. The world report on violence and health. Lancet. 2002;360:1083–8. 12384003 10.1016/S0140-6736(02)11133-0

[3] García-Moreno C, Pallitto C, Devries K, Stöckl H, Watts C, Abrahams N. Global and regional estimates of violence against women: prevalence and health effects of intimate partner violence and non-partner sexual violence. World Health Organization; 2013.

[4] Kalra G, Bhugra D. Sexual violence against women: Understanding cross-cultural intersections. Indian J Psychiatry. 2013;55(3):244–9. doi: 10.4103/0019-5545.117139 24082244 PMC3777345

[5] Heise L, Moore K, Toubia N. Defining “coercion” and “consent” cross-culturally. SIECUS Rep. 1996;24(2):12–4. 12290745

[6] Zambia Statistics Agency M of H (MOH) Z. Zambia demographic and health survey 2018. Lusaka, Zambia, and Rockville, Maryland, USA: Zambia Statistics Agency, Ministry of Health, and ICF. 2019.

[7] Greco D, Dawgert S. Poverty and sexual violence: building prevention and intervention responses. 2007.

[8] Shamu S, Shamu P, Machisa M. Factors associated with past year physical and sexual intimate partner violence against women in Zimbabwe: results from a national cluster-based cross-sectional survey. Glob Health Action. 2018;11(sup3):1625594. doi: 10.1080/16549716.2019.1625594 31232228 PMC6598507

[9] Simona S, Muchindu M, Ntalasha H. Intimate partner violence (IPV) in Zambia: Socio-demographic determinants and association with use of maternal health care. Int’l J Soc Sci Stud. 2018;6:42.

[10] Bengesai AV, Khan HTA. Female autonomy and intimate partner violence: findings from the Zimbabwe demographic and health survey, 2015. Cult Health Sex. 2021;23(7):927–44. doi: 10.1080/13691058.2020.1743880 32285753

[11] Kishor S, Johnson K. Profiling domestic violence: A multi-country study. MEASURE DHS+, ORC Macro; 2004.

[12] Panda P, Agarwal B. Marital violence, human development and women’s property status in India. World Dev. 2005;33(5):823–50.

[13] Kilgallen JA, Schaffnit SB, Kumogola Y, Galura A, Urassa M, Lawson DW. Positive Correlation Between Women’s Status and Intimate Partner Violence Suggests Violence Backlash in Mwanza, Tanzania. J Interpers Violence. 2022;37(21–22):NP20331–60. doi: 10.1177/08862605211050095 34802316

[14] Government of the Republic of Zambia. The Pinal Code Act. 1995.

[15] Government of the Republic of Zambia. Anti-Gender-Based Violence Act. 2011.

[16] Keesbury J, Onyango-Ouma W, Undie C-C, Maternowska C, Mugisha F, Kageha E, et al. A review and evaluation of multi-sectoral response services (‘one-stop centers’) for gender-based violence in Kenya and Zambia. 2012.

[17] Tenkorang EY, Owusu AY, Yeboah EH, Bannerman R. Factors Influencing Domestic and Marital Violence against Women in Ghana. J Fam Viol. 2013;28(8):771–81. doi: 10.1007/s10896-013-9543-8

[18] Alkan Ö, Tekmanlı HH. Determination of the factors affecting sexual violence against women in Turkey: a population-based analysis. BMC Womens Health. 2021;21(1):188. doi: 10.1186/s12905-021-01333-1 33952220 PMC8097900

[19] Memiah PN. Exploring risk factors among female undergraduate college students reporting the experience of sexual violence: A comparative analysis of African Americans and Caucasians. Morgan State University; 2006.

[20] Fischbach RL, Herbert B. Domestic violence and mental health: correlates and conundrums within and across cultures. Soc Sci Med. 1997;45(8):1161–76. doi: 10.1016/s0277-9536(97)00022-1 9381230

[21] Islam MS. Intimate partner sexual violence against women in Sylhet, Bangladesh: some risk factors. J Biosoc Sci. 2022;54(1):54–76. doi: 10.1017/S002193202000067X 33213532

[22] Antai D. Controlling behavior, power relations within intimate relationships and intimate partner physical and sexual violence against women in Nigeria. BMC Public Health. 2011;11:511. doi: 10.1186/1471-2458-11-511 21714854 PMC3161889

[23] Borumandnia N, Khadembashi N, Tabatabaei M, Alavi Majd H. The prevalence rate of sexual violence worldwide: a trend analysis. BMC Public Health. 2020;20(1):1835. doi: 10.1186/s12889-020-09926-5 33256669 PMC7706187

[24] Team RC. R: A language and environment for statistical computing. 2013.

[25] Wickham H, Francois R, Henry L, Müller K. dplyr: A grammar of data manipulation. R package version 04. 2015;3. p. 156.

[26] Bates D, Maechler M, Bolker B, Walker S, Christensen R, Singmann H, et al. Package ‘lme4.’ Linear mixed-effects models using S4 classes R package version. 2011;1.

[27] Goldstein H. Multilevel statistical models. John Wiley & Sons; 2011.

[28] Merlo J, Chaix B, Yang M, Lynch J, Råstam L. A brief conceptual tutorial of multilevel analysis in social epidemiology: linking the statistical concept of clustering to the idea of contextual phenomenon. J Epidemiol Community Health. 2005;59(6):443–9. doi: 10.1136/jech.2004.023473 15911637 PMC1757045

[29] Owoaje ET, OlaOlorun FM. Women at risk of physical intimate partner violence: a cross-sectional analysis of a low-income community in southwest Nigeria. Afr J Reprod Health. 2012;16(1):43–53. 22783667

[30] Fidan A, Bui HN. Intimate Partner Violence Against Women in Zimbabwe. Violence Against Women. 2016;22(9):1075–96. doi: 10.1177/1077801215617551 26644331

[31] Yahaya I, Soares J, De Leon AP, Macassa G. A comparative study of the socioeconomic factors associated with childhood sexual abuse in sub-Saharan Africa. Pan Afr Med J. 2012;11:51. 22593787 PMC3343679

[32] Kimuna SR, Djamba YK, Ciciurkaite G, Cherukuri S. Domestic violence in India: insights from the 2005-2006 national family health survey. J Interpers Violence. 2013;28(4):773–807. doi: 10.1177/0886260512455867 22935947

[33] Oladepo O, Yusuf OB, Arulogun OS. Factors influencing gender based violence among men and women in selected states in Nigeria. Afr J Reprod Health. 2011;15(4):78–86. 22571109

[34] Pandey S. Physical or sexual violence against women of childbearing age within marriage in Nepal: Prevalence, causes, and prevention strategies. Int Soc Work. 2016;59:803–20.

[35] Elder GH Jr. The life course as developmental theory. Child Dev. 1998;69(1):1–12. 9499552

[36] Gage AJ, Hutchinson PL. Power, control, and intimate partner sexual violence in Haiti. Arch Sex Behav. 2006;35(1):11–24. doi: 10.1007/s10508-006-8991-0 16502150

[37] Jejeebhoy SJ. Wife-beating in rural India: a husband’s right? Evidence from survey data. Economic and Political Weekly. 1998;33(15):855–62.

[38] Basile KC, Smith SG. Sexual Violence Victimization of Women. Am J Lifestyle Med. 2011;5(5):407–17. doi: 10.1177/1559827611409512

[39] Diez-Roux AV. Multilevel analysis in public health research. Annu Rev Public Health. 2000;21:171–92. doi: 10.1146/annurev.publhealth.21.1.171 10884951

[40] Turner J, Beeghley L, Powers C. The emergence of sociological theory. Sage Publications; 2011.

[41] Jansen NA, Agadjanian V. Polygyny and Intimate Partner Violence in Mozambique. J Fam Issues. 2020;41(3):338–58. doi: 10.1177/0192513x19876075 33518874 PMC7845931

[42] Ahinkorah BO. Polygyny and intimate partner violence in sub-Saharan Africa: evidence from 16 cross-sectional demographic and health surveys. SSM Popul Health. 2021;13:100729. doi: 10.1016/j.ssmph.2021.100729 33511263 PMC7815814

[43] Heath R, Hidrobo M, Roy S. Cash transfers, polygamy, and intimate partner violence: experimental evidence from Mali. Journal of Development Economics. 2020;143:102410.

[44] Corsi DJ, Neuman M, Finlay JE, Subramanian SV. Demographic and health surveys: a profile. Int J Epidemiol. 2012;41(6):1602–13. doi: 10.1093/ije/dys184 23148108

[45] Simona SJ. Structural violence and maternal healthcare utilisation in sub-Saharan Africa: A Bayesian multilevel analysis. University of Glasgow; 2020.

